# Evaluation of health-related quality of life of female students suffering from primary dysmenorrhea: findings of a cross-sectional study from Pakistan

**DOI:** 10.3389/fpubh.2025.1467377

**Published:** 2025-04-24

**Authors:** Mamoona Dar, Amjad Khan, Syed Sikandar Shah, Ayesha Aleem, Ammar Ali Saleh Jaber, Mulazim Hussain, Gul Majid Khan

**Affiliations:** ^1^Department of Pharmacy, Quaid-i-Azam University, Islamabad, Pakistan; ^2^College of Pharmacy, University of Sargodha, Sargodha, Pakistan; ^3^Department of Pharmacy, The First Affiliated Hospital, Xi’an Jiaotong University, Xi’an, China; ^4^Department of Pharmacy Administration and Clinical Pharmacy, School of Pharmacy, Health Science Center, Xi’an Jiaotong University, Xi’an, China; ^5^Department of Clinical Pharmacy and Pharmacology, RAK College of Pharmacy, RAK Medical and Health Sciences University, Ras Al Khaimah, United Arab Emirates; ^6^Institute of Pharmacy, Gulab Devi Educational Complex, Lahore, Pakistan; ^7^Department of Clinical Pharmacy & Pharmacotherapeutics, Dubai Pharmacy College, Dubai, United Arab Emirates; ^8^Department of Pharmacy, Iqra University, Islamabad, Pakistan

**Keywords:** primary dysmenorrhea, health-related quality of life, university students, cross-sectional study, Pakistan

## Abstract

**Background:**

Primary dysmenorrhea (PD) is a global public health problem affecting the quality of life of menstruating women. This study aimed to assess the health-related quality of life (HRQoL) of female students experiencing PD.

**Methodology:**

This cross-sectional study included 484 female students (aged 16–31 years) from different educational institutes in Sargodha, Pakistan from October 2021 to November 2022. Demographic and menstrual characteristics were collected through interviews using a purpose-developed data collection form, whereas HRQoL was evaluated using the EuroQol-5 Dimensions-5 Level (EQ-5D-5L) questionnaire. Data were analyzed using either Mann–Whitney tests or Kruskal-Wallis analysis of variance with SPSS version 23.

**Results:**

The mean age (SD) of the participants was 22.41 (3.5) years. The majority of participants were aged 21–25 years (58.9%), unmarried (86.2%), had a normal BMI (68.6%), had a family history of PD (58.1%), experienced a regular menstrual cycle (79.8%), and exhibited moderate PD (48.9%). Statistically significant differences in participants’ EQ-5D index scores were observed based on the bleeding duration (*p* = 0.015), the length of the menstrual cycle (*p* = 0.004), cycle regularity (*p* = 0.022), family history (*p* = 0.027) how long the PD symptoms last (*p* < 0.001), and the season in which the PD pain is experienced the most (*p* < 0.001). Moreover, the EQ-VAS score also showed statistically significant differences based on the length of the menstrual cycle (*p* = 0.007), how long the PD symptoms last (*p* < 0.001), and the season in which the PD pain is experienced the most (*p* < 0.001). 51.7% of participants preferred heat application among the various lifestyle modifications to manage PD.

**Conclusion:**

This study indicated that Primary dysmenorrhea (PD) negatively impacts health-related quality of life (HRQoL). Therefore, it is essential to explore effective interventions while raising awareness and improving access to medical care in Pakistan to enhance the HRQoL and well-being of women.

## 1 Introduction

Millions of women of reproductive age face various challenges related to their menstrual cycles, including pain, discomfort, anxiety, feelings of shame, and a sense of isolation (1). The term dysmenorrhea is defined as a condition of menstruation-associated pelvic pain (2). It is classified into two types: primary and secondary dysmenorrhea (3). Primary dysmenorrhea (PD) is defined as spasmodic and painful cramps in the lower abdomen that begin shortly before or at the onset of menses in the absence of any pelvic pathology and radiate to the inguinal area and buttocks (4, 5). Secondary dysmenorrhea is caused by menstrual pain in the presence of underlying organic disease. It could be of either gynecological origin such as endometriosis, myometrial pathologies such as adenomyosis and fibroids, postoperative adhesion syndrome, pelvic inflammatory disease (PID), ovarian cyst, or non-gynecological causes where the pain often results from gastrointestinal diseases or urinary tract diseases (6). PD is more common in adolescent girls and usually appears 6 to 12 months after menarche (3, 7). The severe pelvic pain and cramps in PD are accompanied by headache, vomiting, anorexia, nervousness, breast tenderness, and mood swings before or during periods (8, 9). The precise mechanism of PD remains unknown, but recent studies related it with the increased production and release of prostaglandin and a decrease in the progesterone in the luteal phase resulting in increased contractility of myometrium, uterine muscles ischemia, and decreased pain threshold (10–12). Previous studies reported that high-intensity pain and associated complications in patients with PD negatively affect quality of life resulting in higher rates of absenteeism from educational and professional settings leading to poor performance and productivity compared to their male counterparts (13–16). These challenges related to menstruation are evident as “gender-based obstacles” that result in early low retirement savings (17, 18).

Previous studies demonstrated that PD is a widespread gynecologic problem in young and adult women with a global prevalence of 60–93% (19, 20). However, the prevalence varies due to sociocultural, ethnic, or biological factors in various populations (21). Women afflicted with dysmenorrhea often refrain from seeking medical assistance, and when left unaddressed, it is prone to impede their work performance (10). The higher prevalence percentage in developed countries like Japan and Australia is believed to be linked to poor menstrual literacy (22, 23). In contrast, lower-middle-income countries like Ghana, Nigeria (24), and Pakistan considers menstruation a cultural taboo and a shameful burden (25). Similar neglectful and cultural stigma exists in high-income Asian countries like India and China (26). The countries that include menstrual health and hygiene (MHH) in their health and education agenda seem to focus primarily on period poverty but often neglect serious issues such as debilitating menstrual pain, a decline in mental health, disrupted physical activity, and inability to perform effectively in the workplace (27). So, to conclude despite being a primary health issue of every other menstruating female dysmenorrhea is unaddressed in most countries around the globe.

Some studies have focused on lifestyle modifications (28), MHH (29), pain intensity, and poor academic performance (30). A previous study demonstrated differences in pain severity by 23.5% from severe pain requiring pharmacological intervention to mild and moderate pain categories 23. Similarly, a study investigating lifestyle-focused approaches reported that nutrition, physical activity, Body Mass Index (BMI), herbs, essential oils, and medical plants help manage PD (31). Although Pakistan is the world’s fifth most populated country, no such health-related quality of life (HRQoL) study among menstruating university students has been conducted, even though dysmenorrhea affects almost 78% of these women (32). Furthermore, this age group population is critical to investigate because most of them experience this condition and symptoms at the onset or during their reproductive age. Recognizing the impact of primary dysmenorrhea (PD) on health, quality of life, and daily activities; is crucial (33). Therefore, the present study aimed to investigate the HRQoL of PD menstruating university students, as well as the lifestyle modification approaches used to manage PD.

## 2 Methodology

### 2.1 Study design

A descriptive cross-sectional study was conducted between October 2021 and November 2022 to assess the HRQoL of female students experiencing PD in Sargodha, Pakistan. Participants were recruited from three educational institutions: The University of Sargodha (UoS main campus), Niazi Medical and Dental College, Sargodha, and Doctors Institute of Health Sciences, Sargodha.

### 2.2 Study population

**Figure 1:**
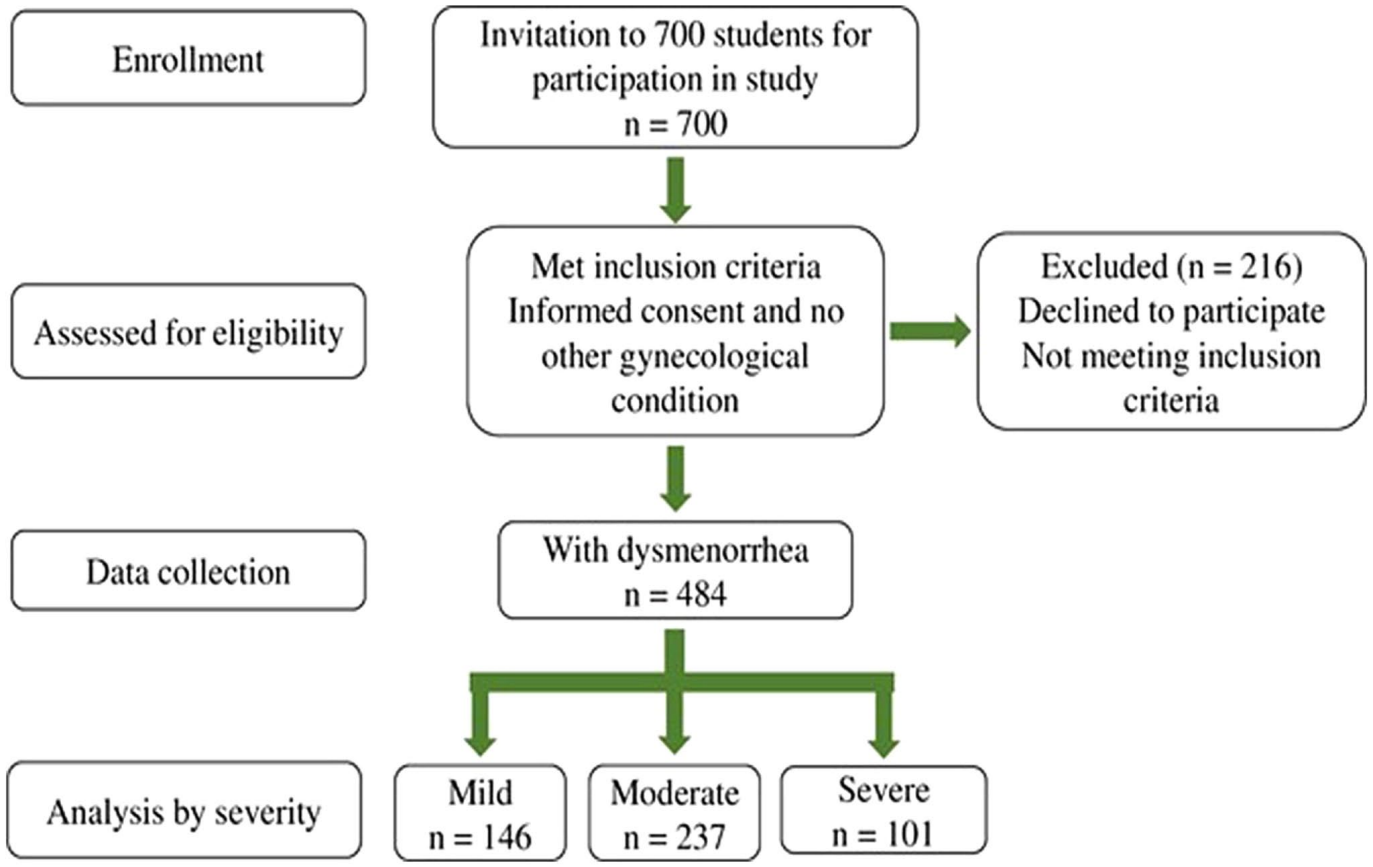
Schematic representation of screening, selection, and methodology used for the participants.

A total of 700 students were contacted to participate in the study. Among them, 484 students met the inclusion criteria: (i) young adult Pakistani females between the ages of 17 and 31 and experiencing PD; (ii) enrolled in either graduation or post-graduation programs; and (iii) with regular menstrual cycles and no gynecological comorbidities. The exclusion criteria included: (i) students who were in academic exchange programs; (ii) students with irregular menstruation or a history of psychological or gynecological illnesses, such as ovarian or cervical cancer, endometriosis, polycystic ovaries, uterine fibroids, or those on hormonal replacement therapy; and (iii) students who did not agree to participate. The subjects who consented to be part of the study but had any diagnosed gynecological pathology or suggestive conditions related to endometriosis, including early menarche, familial history of endometriosis, painful symptoms resisting empirical medical treatment, heavy menstrual bleeding, gastrointestinal and genitourinary symptoms, as well as associated symptoms including nausea, fatigue, and effects on daily activities were excluded from the study. All participants signed a written informed consent form before data collection commenced. A flow chart illustrating the criteria for this study is illustrated in Figure 1.

### 2.3 Participants enrollment and study tools

We used a convenient sampling technique to enroll participants. Of the 700 participants who consented to the study, 484 students met the inclusion criteria. The sampling techniques used were a combination of convenient sampling and simple random sampling. A numerical pain-related scale (NPRS) for pain and visual analogue scale (EQ-VAS) and EQ-5D-5L, were administered to all participants to accurately assess their levels of pain and HRQoL, respectively, (34, 35).

The first part of the questionnaire included demographic information such as age, BMI, place of residence, family history of dysmenorrhea, menstrual cycle regularity, personal feelings about blood flow, and the duration of symptoms of PD. Participants were asked about the season in which they are more vulnerable to pain, the age of menarche, their education level and their subject specialty, and the remedies they use to cope with their menstrual pain. Participants were asked to rate their level of pain using a numerical pain-related scale (NPRS) that ranged from 0 (no pain) to 10 (severe pain). The NPRS is a widely used 11-digit numeric scale for quantifying pain from 0–10. 0 shows no pain, 1–3 for mild pain, 4–6 for moderate pain, and 7–10 shows the worst pain (33).

The EQ-5D-5L questionnaire evaluated the participants’ HRQoL. This questionnaire provides a single index value for health status and a descriptive profile. Using “Euro QoL 5D 5 L,” the health-related quality of life (HRQoL) of patients with primary dysmenorrhea was determined. Participants in the study documented their answers on the visual analogue scale and descriptive section. In the descriptive component, the study participants are asked to choose one level from a set of following five domains—mobility, self-care, usual activity, pain/discomfort, and anxiety/depression (henceforth M, SC, UA, PD, and AD) which best reflects their current state of health. Level 1 is “no problem,” Level 2 is “slight problem,” Level 3 is “moderate problem,” Level 4 is “severe problem,” and Level 5 is “extreme problem/unable to do.” We obtained EQ-5D-5L health states directly from the respondent’s self-reported questionnaire. The EQ-5D-5L measures health states. As a result, EQ-5D-5L has 3,125 potential health states. An EQ-5D-5L score of “11,111″ indicates perfect health, while a score of “55,555” signifies the worst health. The EQ-5D index utility score is derived from England’s EQ-5D-5L value set, as a value set specific to Pakistan was not available (36, 37). The UK value set can be accessed through a link on the EuroQol website^1^ (38). According to the scoring guidelines, an EQ-5D index value of 1 represents the best possible health status, whereas a value of −0.594 indicates the worst health status.

The study participants also selected one point from the 20 cm long visual analogue scale (VAS) to report their perceived health. This scale’s two endpoints are “best imaginable health” and “Worst imaginable health.” The scale has 10 readings from “0” to “100” and the study participants are asked to rate their current health states on this scale (39). If any participant did not understand a question, assistance was provided, and participants were encouraged to answer all questions. The questionnaire took approximately 10–15 min to complete.

### 2.4 Statistical analysis

The initial data sheets were checked, coded, and prepared into Microsoft Excel spreadsheets. The data was then exported to IBM SPSS Statistics version 23.0 (IBM SPSSR Statistics for Windows, version 23.0; IBM Corp., Armonk, NY, United States) for analysis. The HRQoL utility scores (index values) were calculated using the original 1995 UK population data due to a lack of utility score data from the Pakistani population. Our data was not normally distributed after its normality was assessed using the Shapiro–Wilk and Kolmogorov–Smirnov tests. Hence, we performed non-parametric tests. Mann Whitney U and Kruskal Wallis tests wherever applicable were used to evaluate the difference between participants’ EQ-5D-5L and EQ-VAS scores based on their sociodemographic and PD-related characteristics. Findings with a *p* < 0.05 were considered statistically significant.

## 3 Results

### 3.1 Sociodemographic, menstrual, and PD-related characteristics of the study participants

The sociodemographic, menstrual, and PD-related characteristics of study participants are given in Table 1. The mean age (SD) of the study participants was 22.41 (3.5) years. The majority of them were 21–25 years old (58.9%), unmarried (86.2%), urban residents (69.2%), undergraduate students (61%), had a normal BMI (68.6%), family history of PD (58.1%), and regular menstrual cycle (79.8%). According to the WHO standards, Cut-off values for BMI are underweight <18.5, normal 18.50–24.99, overweight ≥25, and obese ≥30.

**Table 1:** Sociodemographic, menstrual, and PD-related characteristics of study participants.

Variable	Frequency	Percent (%)
Age 22.41 (3.5)		
17–20 years	130	26.9
21–25	285	58.9
26–30	41	8.5
31 and above	28	5.8
Marital status		
Single	417	86.2
Married	67	13.8
Residence		
Rural	149	30.8
Urban	335	69.2
Education type		
Health Sciences	395	81.6
Non-Health sciences	89	18.4
Education level		
Undergraduates	295	61
Graduates	180	37.2
Postgraduates	9	1.9
Mother qualification		
Primary	110	22.7
Middle	89	18.4
High school	149	30.8
Graduate or above	136	28.1
BMI		
Underweight	82	16.9
Normal	332	68.6
Overweight	59	12.2
Obese	11	2.3
Age of Menarche		
Younger than 12 years	45	9.3
12-14 years	289	59.7
14 years or older	150	31
Duration of bleeding		
3 days	109	22.5
4 days	164	33.9
5 days	149	30.8
6 days or more	62	12.8
Nature of blood flow		
Heavy	158	32.6
Light	163	33.7
Normal	163	33.7
Menstrual cycle length		
Less than 21 days	57	11.8
22–28 days	295	61
More than 28 days	132	27.3
Cycle regularity		
Regular	386	79.8
Irregular	98	20.2
Spotting between periods		
Yes	206	42.6
No	278	57.4
Family history of PD		
Yes	281	58.1
No	203	41.9
Symptoms of PD last		
Fewer than 3 days	307	63.4
3 days	120	24.8
More than 3 days	57	11.8
Season you experience PD the most		
Summer	101	20.9
Winter	194	40.1
Autumn	3	0.6
Spring	4	0.8
Whole year	182	37.6
Region of pain		
Lower abdomen	434	83.9
Lumber region	40	8.3
Thigh region	10	2.1
Emotional symptoms		
No change in emotional/mood	60	11.6
Feeling angry/irritable for no specific reason	187	36.2
Feeling restless/anxious for no reason	95	19.6
Feeling depressed for no reason	75	14.5
Feeling socially embarrassed	67	13
Physical symptoms		
Ache all over with cold sweating	66	12.8
Bloating	89	17.2
Breast discomfort	42	8.1
Change in appetite	44	8.5
Diarrhea	91	17.6
Constipation	28	5.4
Hot flushes	102	19.7
Combination of more than 2	22	4.3

Moreover, 63.4% of them experienced menstrual pain for less than 3 days, and 32.6% of participants had heavy periods. Interestingly, 59.7% had their first period at 12–14 years. The Numerical Pain Rating Scale (NPRS) was used for pain assessment. On this scale, 30.2% (*n* = 146) had mild PD, whereas 48.9% (*n* = 237) had moderate PD, and 20.9% (*n* = 101) had severe PD, as mentioned in Table 2.

**Table 2:** Prevalence of primary dysmenorrhea mild, moderate, and severe among study populations according to numerical pain-related scale (NPRS).

Status	Severity of pain	Frequency (%)
Primary dysmenorrhea	Mild	146 (30.2)
	Moderate	237 (48.9)
	Severe	101 (20.9)

### 3.2 EQ-5D health status and EQ-VAS score

We observed statistically significant differences in participants’ EQ-5D index scores based on the bleeding duration (*p* = 0.015), the length of the menstrual cycle (*p* = 0.004), cycle regularity (*p* = 0.022), family history (*p* = 0.027), how long the PD symptoms last (*p* < 0.001), and the season in which the PD pain is experienced the most (*p* < 0.001). Moreover, the EQ-VAS score also showed statistically significant differences based on the length of the menstrual cycle (*p* = 0.007), how long the PD symptoms last (*p* < 0.001), and the season in which the PD pain is experienced the most (*p* < 0.001) (Table 3).

**Table 3:** Description of HRQoL scores of demographic and menstrual characteristics.

Variable	N (%)	EQ-5D index score Median values (IQR)	*p*	EQ-VAS Score Median values (IQR)	*p*
Age			0.893**		0.740**
16–20	130 (26.9)	0.217 (−0.074, 0.621)		78 (68–85.25)	
21–25	285 (58.9)	0.516 (−0.074, 0.621)		80 (68.5–85.0)	
26–30	41 (8.5)	0.255 (−0.0075, 0.588)		77 (63.5–85.0)	
31–above	28 (5.8)	0.516 (0.110, 0.678)		77 (65.0–86.0)	
Marital status			0.902*		0.775*
Single	417 (86.2)	0.348 (−0.074, 0.621)		80 (68.0–85.0)	
Married	67 (13.8)	0.516 (−0.074, 0.678)		80 (69.0–86.0)	
Location			0.304*		0.330*
Urban	335 (69.2)	0.348 (−0.042, 0.621)		79 (68.0–85.0)	
Rural	149 (30.8)	0.348 (−0.074, 0.585)		79 (68.0–85.0)	
Education level			0.218**		0.249**
Undergraduate	295 (61)	0.255 (−0.077, 0.585)		79 (67.0–85.0)	
Graduates	180 (37.2)	0.516 (−0.016, 0.621)		80 (69.2–87.7)	
Postgraduate	09 (1.9)	0.621 (−0.193, 0.678)		83 (57.0–86.0)	
Education type			0.140*		0.078*
Health sciences	395 (81.6)	0.348 (−0.074, 0. 592)		79 (67.0–85.0)	
Non-health sciences	89 (18.4)	0.516 (−0.016, 0.678)		80 (70.0–88.0)	
Body mass index			0.136**		0.465**
Underweight	82 (16.9)	0.117 (−0.199, 0.533)		73.5 (62.0–84.2)	
Normal	332 (68.6)	0.516 (−0.0705, 0.621)		80 (69.0–85.0)	
Overweight	59 (12.2)	0.516 (−0.016, 0.621)		80 (68.0–85.0)	
Obese	11 (2.3)	0.348 (−0.042, 0.0.621)		75 (67.0–90.0)	
Mother’s education			0.532**		0.276**
Primary	110 (22.7)	0.255 (−0.074, 0.599)		78.5 (67.5–85.0)	
Middle	89 (18.4)	0.348 (−0.119, 0.621)		75 (68.0–85.0)	
High school	149 (30.8)	0.516 (−0.16, 0.592)		80 (70.0–85.0)	
Graduate and above	136 (28.1)	0.255 (−0.074, 0.62)		75 (65.0–85.0)	
Age of menarche			0.670**		0.930**
< 12 years	45 (9.3)	0.077 (−0.115, 0.621)		78 (65.0–85.0)	
12–14 years	289 (59.7)	0.516 (−0.042, 0.592)		80 (69.0–85.0)	
14 years or older	150 (31)	0.348 (−0.074, 0.621)		79.5 (67.5–85.2)	
Menstrual cycle length			0.004**		0.007**
< 21 days	57 (11.8)	0.077(−0.212, 0.516)		70 (61.5–81.0)	
22–28 days	295 (61)	0.516(−0.016, 0.621)		80 (70.0–85.0)	
> 28 days	132 (27.3)	0.301 (−0.074, 0.613)		79 (67.2–85.0)	
Blood flow			0.224**		0.255**
Heavy	158 (32.6)	0.157 (−0.074, 0.585)		78 (65.0–85.0)	
Light	163 (33.7)	0.516 (−0.115, 0.621)		77 (69.0–85.0)	
Normal	163 (33.7)	0.0516 (−0.016, 0.678)		80 (70.0–85.0)	
Cycle regularity			0.022*		0.056*
Regular	386 (79.8)	0.516 (−0.042, 0.621)		80 (69.0–85.0)	
Irregular	98 (20.2)	0.077 (−0.115, 0.585)		75 (65.0–85.0)	
Family history			0.027*		0.980*
Yes	281 (58.1)	0.180 (−0.074, 0.585)		78 (67.5–85.0)	
No	203 (41.9)	0.516 (0.001, 0.621)		80 (69.0–86.0)	
Spotting between periods			0.228*		0.306*
Yes	206 (42.6)	0.255 (−0.0803, 0.621)		77.5 (69.0–85.0)	
No	278 (57.4)	0.516 (−0.0225, 0.621)		80 (68.0–85.0)	
Bleeding duration			0.015**		0.121**
3 days	109 (22.5)	0.516 (−0.016, 0.693)		80 (70.0–86.0)	
4 days	164 (33.9)	0.516 (−0.066, 0.621)		77.5 (68.0–85.0)	
5 days	149 (30.8)	0.157 (−0.115, 0.533)		79 (65.0–85.0)	
6 or more days	62 (12.8)	0.217 (−0.074, 0.558)		78 (69.0–84.2)	
Symptoms of PD last for			<0.001**		<0.001**
< 3 days	307 (63.4)	0.516 (0.001, 0.678)		80 (70.0–86.0)	
3 days	120 (24.8)	0.168 (−0.074, 0.553)		78 (65.0–84.7)	
> 3 days	57 (11.8)	−0.074 (−0.199, 0.255)		70 (60.5–80.0)	
The season you experience PD the most			<0.001**		<0.001**
Summer	101 (20.9)	0.516 (−0.035, 0.678)		80 (69.5–87.5)	
Winter	194 (40.1)	0.516 (0.046, 0.678)		81 (70.0–86.0)	
Autumn	03 (0.6)	0.077 (0.335, 0.)		61 (52.0–62.0)	
Spring	04 (0.8)	0.73 (0.728, 0.781)		92.5 (82.5–95.0)	
Whole year	182 (37.6)	0.077 (−0.199, 0.516)		75 (62.–81.2)	

*Mann–Whitney U test; **Kruskal Wallis test; *p* < 0.05 shows a statistically significant difference.Bold values display the *p*-value of statistical significance.

### 3.3 EQ-5D-5L dimensions

Table 4 presents the percentage distribution of five health dimensions among PD participants. Our study found that the most frequently reported issue among these dimensions was with usual activities, where approximately 40.9% of females experienced moderate problems. In addition, 34.1% reported moderate impairment in mobility, and 27.9% faced moderate issues with self-care. Notably, 33.5% of participants reported experiencing extreme pain or discomfort, while 26.2% indicated being extremely anxious or depressed, as illustrated in Table 4.

**Table 4:** Responses to health-related quality of life questions.

Sr No.	Domains	*N*	Percentage (%)
1	Mobility		
	I have no problems walking about	150	31
	I have slight problems in walking about	53	11
	I have moderate problems in walking about	165	34.1
	I have severe problems in walking about	61	12.6
	I am unable to walk	55	11.4
2	Self-care		
	I have no problems washing or dressing myself	124	25.6
	I have slight problems washing or dressing myself	109	22.5
	I have moderate problems washing or dressing myself	135	27.9
	I have severe problems washing or dressing myself	60	12.4
	I am unable to wash or dress myself	56	11.6
3	Usual activities		
	I have no problems doing my usual activities	183	37.8
	I have slight problems doing my usual activities	9	1.9
	I have moderate problems doing my usual activities	196	40.5
	I have severe problems doing my usual activities	39	8.1
	I am unable to do my usual activities	57	11.8
4	Pain/Discomfort		
	I have no pain or discomfort	0	0
	I have slight pain or discomfort	73	15.1
	I have moderate pain or discomfort	212	43.8
	I have severe pain or discomfort	37	7.6
	I have extreme pain or discomfort	162	33.5
5	Anxiety/Depression		
	I am not anxious or depressed	39	8.1
	I am slightly anxious or depressed	47	9.7
	I am moderately anxious or depressed	223	46.1
	I am severely anxious or depressed	48	9.9
	I am extremely anxious or depressed	127	26.2

Percentage distribution of EQ-5D-5L domains.

### 3.4 Measures adopted for pain management

Different lifestyle modification methods were employed for relieving menstrual pain in females. This study revealed that among all participants 51.7% (*n* = 250) were using heat application method either via hot water bottle or heating patches to relieve their pain, whereas 5.4% (*n* = 26) tried to divert their attention by watching television and 5.6% (*n* = 27) listening to music. 10.3% (*n* = 50) opted for a massage to relieve the pain. Only 1% (*n* = 05) considered meditation and yoga while hot tea was used by 2.7% (*n* = 13) females. Moreover, 5.6% (*n* = 27) chose walking as a pain relief strategy, and 7% % (*n* = 34) preferred endurance. The remaining 9.7% (*n* = 47) use sleep as a coping strategy to get comfort during dysmenorrhea as shown in Table 5.

**Table 5:** Pain relief methods employed by participants.

Pain relief methods	Frequency (N)	Percentage (%)
Heat application	250	51.7%
TV	26	5.4%
Music	27	5.6%
Massage	50	10.3%
Meditation	05	1%
Yoga	05	1%
Hot drinks	13	2.7%
Walk	27	5.6%
Endurance	34	7%
Sleep	47	9.7%

## 4 Discussion

The objective of this study is to examine how the severity of dysmenorrhea affects the health-related quality of life (HRQoL) among female university students in Pakistan. The study reveals a strikingly high prevalence of PD, which may be attributed to a lack of menstrual literacy. This contributes to the baseline data, shedding light on the emotional and physical challenges that Pakistani women face every month, resulting in poor HRQoL across all five domains of EQ-5D-5L: mobility, self-care, usual activities, depression/anxiety, and pain/discomfort. Through the exploration of the relationship between PD and HRQoL of female university attendees, this study uncovers notably a high prevalence of dysmenorrhea, reaching 91.5% which closely aligns with findings from a previous study conducted in a university in Ireland (40), and is comparable to rates observed in King Saud University (80.1%) and females in Nigeria (69.8%) (41, 42). Similarly, the prevalence of PD among Brazilian women was found to be 90.7% in the last menstrual cycle considering mild moderate, and severe pain levels (43). The mean age of the students selected for this study was 22.4 ± 3.5 years, almost similar to an Indian study where the mean age was India (21.99 ± 3.75 years) (44) as Pakistan and India share a close culture and ethnicity. Though Pakistan also shares a close boundary with China the mean age (19.0 ± 1.2 years) was quite different from our study results (20).

From the EQ-5D-5L dimensions, 34.1% experienced moderate impairment in mobility. At the same time, 27.9% reported having moderate problems with self-care. It is worth noting that about 40.9% of females reported moderate problems with usual activities 33.5% of the participants reported extreme pain or discomfort, and 26.2% reported being extremely anxious/depressed, as shown in Table 4.

Regarding pain severity, 30.2% of women in our study reported mild pain, which aligns closely with the reported prevalence among Japanese adult females (33.1%) (13). However, for moderate and severe pain, our study revealed 48.9% moderate and 20.8% reporting severe pain closely following a Saudia study where participants were having moderate 50%, and severe pain 27%, respectively, (41). Overall, the findings from our study suggested that dysmenorrhea significantly impacts the quality of life, affecting not only physical aspects such as mobility and self-care but also influencing usual activities and contributing to pain, discomfort, anxiety, and depression. Additionally, the *p-value* revealed that EQ-5D index scores were significantly associated with the family history (*p* = 0.027), cycle regularity (0.022), bleeding duration (0.015), the length of the menstrual cycle (0.004), how long PD symptoms last (< 0.001), and the season in which you experience pain the most (< 0.001). These results were consistent with previously reported literature (3) (see Table 5).

In our study, the prevalence rates of challenges reported in mobility (67.9%), personal care (70.7%), and daily activities (60.3%) among participants. Another study conducted by Fernández-Martínez et al. (45), reported mobility problems (3%), personal care problems (0.7%), manifested problems regarding daily activities, (5.3%) discomfort/pain (16.5%), and 24.2% had problems related to anxiety/depression. Statistical significance was noted between these dimensions and dysmenorrhea in our study, with a *p*-value of <0.001 for all dimensions of the HRQol scale. The findings of a Spanish study revealed significant differences regarding problems in the dimension of pain and discomfort in women with dysmenorrhea (*p* = 0.036). However, for the remaining dimensions, statistically significant differences were not found (45). In Jordanian women, dysmenorrhea was found to adversely affect university performance and social attitudes toward family and friends (46). Most female medical students in Saudia Arabia suffer from PD, which adversely affects their quality of life and academic performance (41).

The study further disclosed that 51.7% of females experiencing severe pain use heat application as a method for pain relief, consistent with findings in Ireland (79%) and Spain (68.6%) (45). The same practice of sleep and the application of warm objects on the abdomen was used by midwife trainees and nurses in Ghana to reduce pain (47).

In contrast, a notable 41.8% of Nigerians use the hot water bottle method (42), hot tea was common among Japanese females with severe pain (13). Among other non-pharmacological pain-relieving methods, massage was selected by (10.3%) of our study respondents, whereas 25.9% were reported by a Nigerian study (42). Additionally, only 1% preferred yoga as compared to 5.2% of Spanish females who opted for yoga (45). Interestingly Japanese females seem not inclined toward yoga (13).

Numerous studies reveal that Engaging in exercise, which encompasses endurance and various physical activities, has been recognized as beneficial in managing menstrual pain. In our study, 7% of participants chose endurance, and 9.7% preferred sleep, while in another study almost 8% of individuals experiencing severe and moderate pain utilized mind–body medicine, such as endurance activities and 11% of those with moderate pain addressed their discomfort through sleep (48).

For females who prefer to use analgesics, medical treatment with NSAIDs is advised as the first-line therapy (49–51). They should be started one to 2 days before menstruation, taken with meals to reduce gastrointestinal side effects, followed on a regular dosage schedule, and continued for the first 2 to 3 days of bleeding to achieve the best possible treatment efficacy and safety (50–52). Acetaminophen is a safe analgesic alternative with manageable gastrointestinal side effects; it decreases prostaglandin synthesis and has a mild COX inhibitory action (53). Therefore, acetaminophen is only recommended for mild to moderate dysmenorrheic pain (4). Female patients who do not respond to NSAIDs, hormone-based therapies, or non-pharmacological treatments are considered (54). Levonorgestrel intrauterine systems, subcutaneous depot medroxyprogesterone acetate, combined oral contraceptives (COCs), contraceptive transdermal patches or vaginal rings, and other hormonal therapy methods are effective in treating PD (4).

We employed the EQ-5D-5L questionnaire to assess the impact of dysmenorrhea on QoL among female university attendees, the same instrument was used in a study conducted in Spain (45). The validated questionnaire was selected because it provides a standardized method for assessing health-related quality of life (HRQoL) across different health conditions (55). Also, there is a substantial body of research supporting the validity, reliability, and responsiveness of the EQ-5D-5L questionnaire, making it a trusted tool in health outcomes research (56). Other investigations have utilized various questionnaires such as the quality of life enjoyment and satisfaction questionnaire (Q-LES-Q-SF) (57), Short Form-36 (SF-36) (58), and health-related QoL questionnaire WHO/QOL-26 (59). Regardless of the specific scale used, findings consistently indicate a significant impairment in the HRQoL of females experiencing issues with dysmenorrhea across all or certain dimensions of the respective scales.

Menstrual hygiene management (MHM) is a socially taboo topic in Pakistan. Students and teachers have limited access to information about it, MHM (menstrual health and hygiene) is not covered in the curriculum, and Pakistan’s educational system lacks policy guidelines for MHM practices (60). PD should not be seen as a barrier; instead, it can serve as an incentive to research effective solutions for this complaint. This includes exploring both individual treatments and combinations with therapies that have already been validated in scientific studies. In this regard, researchers should aim for consensus while planning their investigations and use reliable methods to improve quality of life.

## 5 Conclusion

The current study found that PD negatively impacts the overall health-related quality of life (HRQoL) of university students, adversely affecting both their mental and physical health. This research marks the very first attempt in Pakistan to evaluate the HRQoL of females who had complaints of PD, acknowledging the significant influence of lifestyle and cultural background on women’s health and perception of pain.

### 5.1 Limitations of study

There are certain limitations of the study which should be considered. Although, it is the first study to find the relationship between dysmenorrhea and HRQoL in a group of Pakistani university students, however, we recommend that more research should be conducted in diverse study settings. The study followed a cross-sectional design, implying that the time of the month when participants were asked to complete the questionnaire could potentially influence their results. A numerical pain-related scale (NPRS) has been used to identify the extent of pain and thereby, the classification of pain as mild, moderate, or severe, was solely based on the patient’s understanding of the severity of pain. Despite these limitations, our study is the first to investigate the HRQoL of Pakistani university students. These findings contribute to the development of knowledge on how young women with PD struggle.

### 5.2 Future recommendations and implications for research

The current study’s strength is that it is the first to evaluate the health-related quality of PD patients. It will assist governments and policymakers in implementing MHH and gaining a profound understanding of the significance of women’s suffering. Researchers should prioritize the need for effective interventions, along with promoting awareness, establishing new accessible treatment options, and facilitating access to medical attention in Pakistan to alleviate the effects of PD and enhance the well-being of women. It is crucial to acknowledge the physical and emotional challenges faced by Pakistani females with dysmenorrhea, recognizing these as gender-based obstacles contributing to poor occupational and economic loss due to women’s absenteeism at work. It necessitates a deep understanding of the significance of MHH by governments and policymakers. In Pakistan to achieve sustainable goals in health, wellbeing, education, and gender equality it requires addressing the gap in MHH. By emphasizing the need for comprehensive policies that address educational and societal aspects related to menstruation to counter menstrual taboo and stigma and improve the overall well-being of women facing menstrual health challenges.

## Data Availability

The raw data supporting the conclusions of this article will be made available by the authors, without undue reservation.

## Footnotes

^1^ www.euroqol.org
